# Synergistic effect of paclitaxel and epigenetic agent phenethyl isothiocyanate on growth inhibition, cell cycle arrest and apoptosis in breast cancer cells

**DOI:** 10.1186/1475-2867-13-10

**Published:** 2013-02-07

**Authors:** Katherine Liu, Shundong Cang, Yuehua Ma, Jen Wei Chiao

**Affiliations:** 1 Department of Medicine, New York Medical College, Valhalla, NY 10595, USA; 2 Department of Oncology, Henan Province People’s Hospital, Zhengzhou, China

## Abstract

This study examined whether combining paclitaxel (taxol) with a novel epigenetic agent phenethyl isothiocyanate (PEITC) will yield a synergistic effect on inhibiting breast cancer cells. Two drug-resistant breast cancer cell lines, MCF7 and MDA-MB-231, were treated with PEITC and taxol. Cell growth, cell cycle, and apoptosis were examined. The combination of PEITC and taxol significantly decreased the IC_50_ of PEITC and taxol over each agent alone. The combination also increased apoptosis by more than two fold over each single agent in both cell lines. A significant increase of cells in the G2/M phases was detected. In conclusion, the combination of PEITC and taxol exhibits a synergistic effect on growth inhibition in breast cancer cells. This combination deserves further study *in vivo*.

## Introduction

Two common epigenetic regulations are DNA methylation and histone acetylation, which modify DNA and histone interactions within chromatins and account for the increase or decrease in gene expression [1-3]. DNA hypermethylation has been shown to inhibit gene transcription, thus reducing gene expression [4-7]. Methylation and deacetylation have been found to play a key role in malignant disorders [8]. Inhibitors of these processes, such as methyltransferase inhibitors and histone deacetylase (HDAC) inhibitors, are novel anti-cancer agents. Two DNA methyltransferase inhibitors, azacitidine and decitabine, and a histone deacetylase inhibitor, vorinostat, have been licensed for clinical use [9-11]. Phenethyl isothiocyanate (PEITC) belongs to the family of natural isothiocyanates, which are found in a wide variety of cruciferous vegetables, and are released when the vegetables are cut or masticated. PEITC has been proven to be an effective HDAC inhibitor, and is able to induce growth arrest and apoptosis in cancer cells both in vitro and in vivo [12-15].

Breast cancer is the most commonly diagnosed cancer among women, accounting for more than 1 in 4 cancers [16]. After lung cancer, breast cancer is the leading cause of cancer death in women. Chemotherapy is a mainstay in breast cancer therapy. New agents are being actively sought [17-21]. Paclitaxel (taxol) is a widely used chemotherapy drug in the treatment of breast cancer [22], lung cancer [23], and ovarian cancer [24]. It was first discovered in 1967 [25], entered clinical trials in 1984 [26-28], and has been a leading chemotherapeutic agent ever since [23,26,27,29]. The mechanism of action of paclitaxel involves its interference with microtubule assembly [30]. Paclitaxel prevents the disassembly of microtubules during mitosis [31]. When taxol binds to tubulin, the microtubules become locked in polymerized state, and thus the cells are restricted from G2 to M phase transition [32-35]. The end result is that the cells are not able to replicate. Another effect of taxol is that it inhibits the anti-apoptosis protein Bcl-2, and induces apoptosis in cancer cells [36]. However, paclitaxel, like most other chemotherapy drugs, has a high level of toxicity as well as a multitude of side effects. The consequence of the toxicity of taxol at a higher dosage is neuropathy which limits its use in patients [23,26,27]. Furthermore, cancer cells develop resistance to taxol after prolonged use.

It has been shown in this laboratory that PEITC is a HDAC inhibitor and can suppress HDAC enzyme activity and decrease HDAC enzyme expression in prostate cancer, leukemia, and myeloma cells [12-14,37-40]. An interesting is that some isothionates have minimal toxicity to normal cells [40]. This project aimed to study the combined effect of PEITC and taxol on breast cancer.

## Materials and methods

### Chemicals and cell cultures

The PEITC (phenethyl isothiocyanate) was purchased from LKT Labs with 98% purity. The PEITC was in Paclitaxel (taxol) powder (Sigma Chemical Co.) was dissolved in DMSO to a stock concentration of 200 nM.

The MCF7 and MDA-MB-231 cell lines were obtained from American Type Cell Cultures. The cells were seeded at 0.4 × 10^6^ per ml and 0.2 × 10^6^ per ml, respectively, of PRMI-1640 medium supplemented with 10% heat-inactivated fetal bovine serum and maintained at 37 C in a humidified atmosphere containing 5% CO_2_. The cells in exponential growth were exposed to PEITC and taxol at various concentrations. The control cultures were supplemented with DMSO as the vehicle control. At the specified time points, the cells were harvested. Cell number and viability were determined from at least triplicate cultures by the trypan blue exclusion method.

### Cell cycle analysis

The analysis of cell cycle phases was performed using a Becton-Dickinson FACScan flow cytometer according to the methods described previously [40]. The cells were stained with propidium iodide solution (50 μg/ml) on ice, and at least 10,000 cells were analyzed.

### Apoptosis analysis

Apoptotic cells were determined by the terminal deoxynucleotidyl transferase-mediated biotinylated UTP nick-end labeling (TUNEL) assay. The TUNEL assay, according to the methods described previously [40], was performed *in situ* with a cell death detection kit (Roche Diagnostics). To enumerate the apoptotic cells, six different fields on each section were examined. At least 100 cells from each field were counted. The mean populations of apoptotic cells per section from the control group and experimental group were reported.

### Statistical analysis

Results from 3 of more experiments were analyzed and expressed as the mean +/- SD. Results were evaluated by a two-sided paired Student’s *t*-test for statistical difference between treatments. *P* <0.05 was considered to be statistically significant. IC_50_, the concentration at which 50% of cell growth is inhibited, was calculated using the Calcusyn software (Biosoft, Inc). Synergism was assessed by the dose–effect curves of single versus combined drug treatment using the Calcusyn software [41].

## Results

### Effect of PEITC and taxol on breast cancer cells

**Figure 1:**
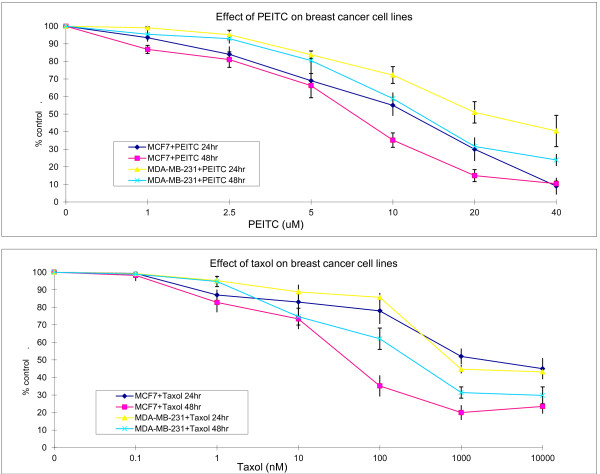
**Single agent phenethyl isothiocyanate (PEITC) and paclitaxel (taxol)-induced growth inhibition of MCF7 and MDA-MB-231 breast cancer cells. **The cells were treated with PEITC or taxol at various concentrations. The viable cells were enumerated at 24 and 48 hours, respectively. Vertical bars represent the means +/- SD of 3 or more independent experiments

To test the effect of PEITC and taxol on breast cancer cells, the agents were added to the MCF7 (MCF) and MDA-MB-231 (MB) cell cultures at serial dilutions for 24 and 48 hours, respectively. The PEITC concentration ranged from 1 to 40 μM (Figure 1), and taxol concentration ranged from 0.1 to 10,000 nM (Figure 1). PEITC suppressed cell growth in a time- and concentration-dependent manner. The IC_50_ (the concentration at which 50% of cell growth is inhibited) of PEITC for MCF cells at 48 hours is 5.6 μM, the IC_50_ of PEITC for MB cells at 48 hours is 15.6 μM. It appears that 5 μM and 10 μM are the concentrations that can cause growth suppression in a linear fashion for MCF and MB cells, respectively. These concentrations were therefore chosen for further combination studies. The IC_50_ of taxol for MCF and MB cells at 48 hours is 111 nM and 410 nM, respectively. The 10 nM and 100 nM concentrations of taxol were chosen for further combination studies for MCF and MB cells, respectively. It appears that MB cells are more resistant to PEITC (>2 x IC_50_) and taxol (4x IC_50_) than MCF cells, and higher concentrations of taxol did not further enhance the effect on growth inhibition.

### Effect of PEITC and taxol in combination on breast cancer cell growth

**Figure 2:**
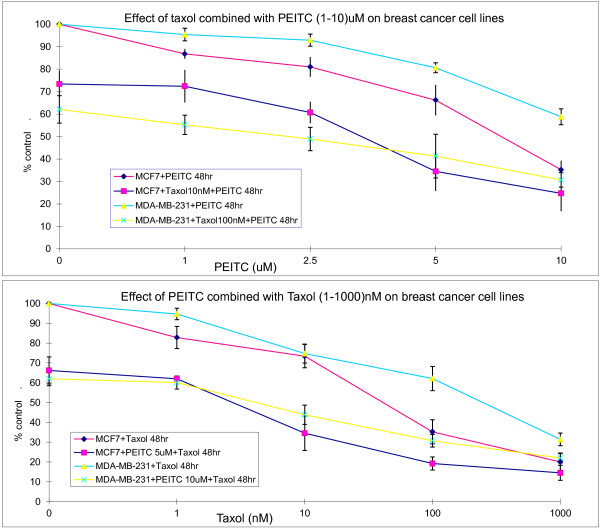
**Effect of combination of PEITC and taxol on growth inhibition of MCF7 and MDA-MB-231 breast cancer cells. **The cells were treated with fixed concentration of PEITC and various concentrations of taxol or vice versa for 48 hours. The viable cells were enumerated at the end of the treatment. Vertical bars represent the means +/- SD of 3 or more independent experiments

**Figure 3:**
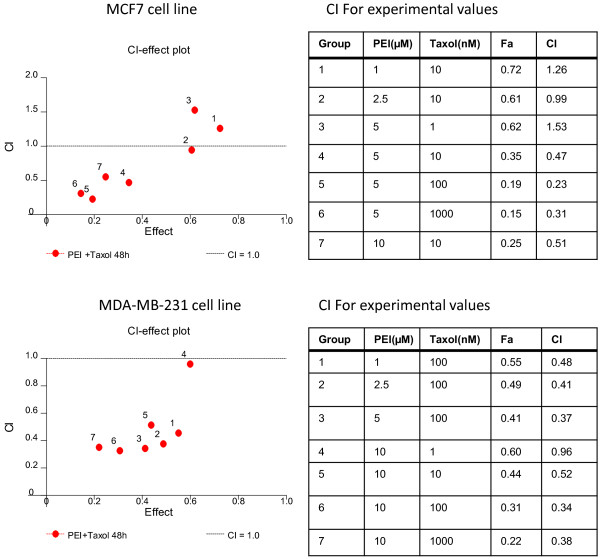
**Combination dose-effect of PEITC and taxol on MCF7 and MDA-MB-231 breast cancer cells. **Cells were cultured in the presence of PEITC and taxol at various concentrations as indicated in the tables for 48 hours. The number of cells were then enumerated. The dose-effect plot is done using Calcusyn software. CI: combination index. Fa: fraction affected; Index >1.0 indicates antagonism, index <1.0 indicates synergism

We further tested the effect of the combination of the two agents on breast cancer cell growth at 48 hours. To search for the optimal concentrations of the two agents, various concentrations were tested. When cells were treated with a fixed concentration of taxol, IC_50_ of PEITC for MCF and MB cells decreased by more than 2.6 folds and 7.3 folds, respectively (Figure 2). When the cells were treated with a fixed concentration of PEITC, the taxol IC_50_ for MCF and MB cells decreased by more than 37 folds and 50 folds, respectively (Figure 2). This effect was further analyzed for synergism using computer modeling. For both MCF and MB cells, there is a clear synergistic effect when PEITC and taxol are combined, although antagonistic effects were seen in certain combinations (Figure 3).

### Effect of combination of PEITC and taxol on cell cycle in breast cancer cells

**Figure 4:**
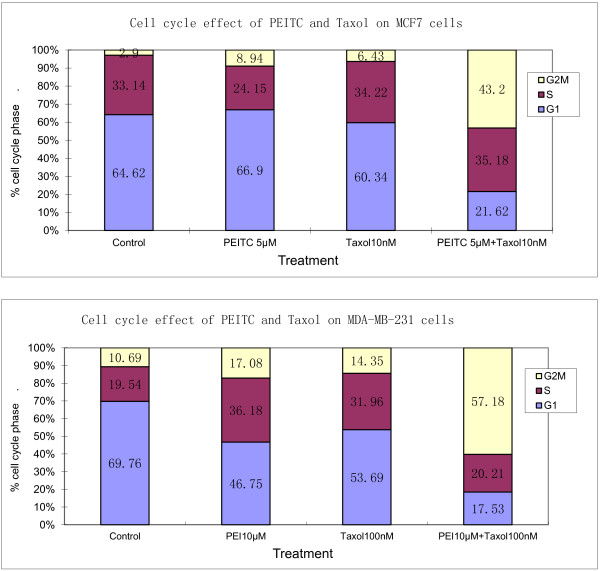
**PEI and taxol combination increases G2M cell cycle arrest in MCF7 and MDA-MB-231 breast cancer cells. **The MCF7 cells were treated with 5 μM of PEITC and 10 nM of taxol alone or in combination. Due to higher resistance of MDA-MB-231 cells, PEITC at 10 μM and taxol at 100 nM were used. The DNA content was by flow cytometry to determine the distribution of cells in each phases

It is known that taxol can suppress cell growth through blocking cell cycle arrest at G2M phases. We therefore examined the effect of combining both agents on cell cycle progression. Taxol and PEITC as single agent at low concentrations caused an accumulation of cells in G2M (PEITC: 8.94% and 17.08%; taxol: 6.43% and 14.35%, for MCF and MB, respectively) (Figure 4). When PEITC and taxol were added concurrently in the cell culture for 48 hours , there was a significant increase (43.2% and 57.1% for MCF and MB, respectively) in the number of cells arrested in the G2M phases (*P* < 0.001) and a corresponding decrease of cells in the G1 phases (*P* < 0.001).

### Effect of combination of PEITC and taxol on apoptosis of breast cancer cells

**Figure 5:**
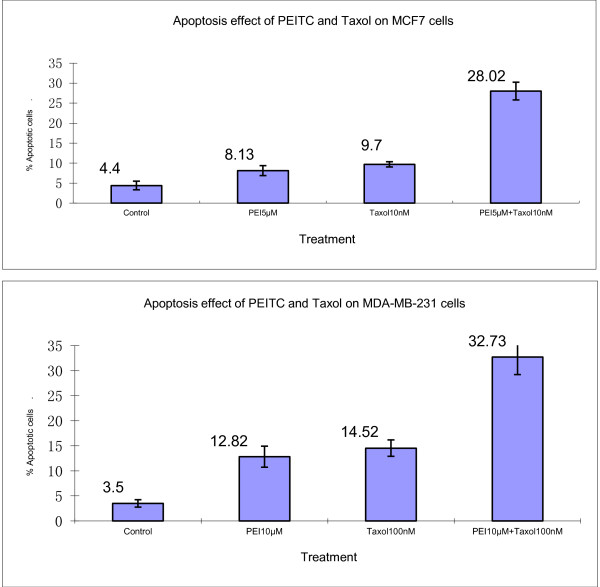
**PEI and taxol combination increases apoptosis of MCF7 and MDA-MB-231 breast cancer cells. **The MCF7 cells were treated with 5 μM of PEITC and 10 nM of taxol alone or in combination. Due to higher resistance of MDA-MB-231 cells, PEITC at 10 μM and taxol at 100 nM were used. Apoptosis was determined using TUNEL assay as described in Materials and Methods. Vertical bars represent the means +/- SD of 3 independent experiments

Using TUNEL assay, the effect of PEITC and taxol on cell apoptosis was examined. Compared with either agent alone, the combination of PEITC and taxol increased apoptosis by 3.4- and 2.8- folds, respectively, in MCF cells, and by more than two folds in MB cells (Figure 5).

## Discussion

Paclitaxel has been a major chemotherapeutic agent for breast cancer and a variety of solid tumors [22-24]. Its major clinical limitations are neurotoxicity and cellular resistance after prolonged treatment. PEITC is a novel epigenetic agent with a dual effect of histone deacetylation and DNA methylation [13,14]. This study found that the two agents have a profound synergistic inhibitory effect on the growth of two different breast cancer cell lines, MCF and MDA-MB-231. The IC_50_ of PEITC and taxol decrease dramatically when the two chemicals are used in combination. These results suggest that it is highly possible to significantly reduce side effects of taxol while maintaining or enhancing clinical efficacy by combining the two drugs.

We hypothesize that by combining PEITC and taxol, it is possible to significantly reduce toxicity *in vivo* by reducing the dosage of taxol needed while maintaining clinical efficacy for breast cancer and other solid tumors. This hypothesis appears to be supported by this in vitro study, and can be tested further in mouse model carrying breast cancer xenografts.

Novel agents targeting different molecular pathways are being actively studied for targeted cancer therapy [18,21,42-46]. A recent study has shown that the HDAC inhibitor vorinostat can up-regulate estrogen receptors and make breast cancer cells more sensitive to tamoxifen [47]. A preliminary report from a recent clinical study seems to corroborate this laboratory finding, where patients with hormone-refractory breast cancer showed responses to tamoxifen again after vorinostat treatment [48]. Since PEITC is a HDAC inhibitor as well as a tubulin-targeting agent, it would be worthwhile to test the combination of PEITC and tamoxifen for therapy of hormone-refractory breast cancer.

Similar to previous reports, we also observed that very high concentrations of taxol did not further increase growth inhibition and apoptosis. This may be due to the fact that higher concentrations of taxol have the opposite effect on cell growth as reported earlier [49]. The exact mechanism remains unclear.

In conclusion, this is the first study to show that the combination of the epigenetic agent PEITC with the chemotherapeutic agent taxol exhibits a synergistic effect on growth inhibition, cell cycle arrest, and apoptosis in breast cancer cells. This novel strategy deserves further study *in vivo*.

## Competing interests

The authors have no relevant conflicts of interests.

## Authors’ contributions

KL, SC and YM performed laboratory studies. All authors have contributed to data preparation, drafting and revising the manuscripts. All authors have read and approved the final manuscript.
